# Medical Specialties

**Published:** 1886-09

**Authors:** 


### MEDICAL SPECIALTIES.

Dr. Wardlaw, the President of the Southern Dental Association, in his annual address, very ably discussed the relation of dentistry to medicine, and came to the conclusion that the former is necessarily a specialty of the latter.

There is a view of this question which dentists seldom consider. How can a man regularly practice a specialty in medicine if he is not himself a medical man? Ophthalmologists, dermatologists, etc., are medical men, holding a regular medical degree. If they do not possess this, the distinctive diploma of medicine, they certainly are not and cannot be recognized as practicing anything *in medicine,* whether a specialty or otherwise.

